# Effects of Growth and Mutation on Pattern Formation in Tissues

**DOI:** 10.1371/journal.pone.0048772

**Published:** 2012-11-07

**Authors:** Benedicte Mengel Pers, Sandeep Krishna, Sagar Chakraborty, Simone Pigolotti, Vedran Sekara, Szabolcs Semsey, Mogens H. Jensen

**Affiliations:** 1 Center for Models of Life, Niels Bohr Institute, University of Copenhagen, Copenhagen, Denmark; 2 National Centre for Biological Sciences, Tata Institute of Fundamental Research, Bangalore, India; Lund University, Sweden

## Abstract

In many developing tissues, neighboring cells enter different developmental pathways, resulting in a fine-grained pattern of different cell states. The most common mechanism that generates such patterns is lateral inhibition, for example through Delta-Notch coupling. In this work, we simulate growth of tissues consisting of a hexagonal arrangement of cells laterally inhibiting their neighbors. We find that tissue growth by cell division and cell migration tends to produce ordered patterns, whereas lateral growth leads to disordered, patchy patterns. Ordered patterns are very robust to mutations (gene silencing or activation) in single cells. In contrast, mutation in a cell of a disordered tissue can produce a larger and more widespread perturbation of the pattern. In tissues where ordered and disordered patches coexist, the perturbations spread mostly at boundaries between patches. If cell division occurs on time scales faster than the degradation time, disordered patches will appear. Our work suggests that careful experimental characterization of the disorder in tissues could pinpoint where and how the tissue is susceptible to large-scale damage even from single cell mutations.

## Introduction

Tissues and organs of eukaryotic organisms develop by cell division and cellular differentiation. While organs exhibit a great variety in their morphology, tissues can often be approximated by a two-dimensional layered structure. Such tissues can be modeled on a hexagonal 2D lattice [1]–[3].

Cellular differentiation involves switching between patterns of gene expression, and is often controlled by signals produced by other cells of the organism. In general, cells can receive signals over large distances (endocrine signaling) [4], short distances (paracrine signaling) [5], or via direct contact of neighboring (juxtacrine signaling) or distant cells (e.g. through tunneling nanotubes) [6]. Direct contact allows for very precise control of cell differentiation because it does not require diffusion of a chemical signal in the intercellular space.

Juxtacrine signalling pathways are present in many multicellular organisms, and can be implemented by different mechanisms [7]–[10]. Using juxtacrine signaling, cells expressing a signal can influence the gene expression pattern of their neighboring cells. Such mechanisms may result in two distinct differentiation pathways in the initially equivalent cells, allowing formation of fine-grained patterns in larger structures [1], [11].

The example of juxtacrine signaling inspired the study of patterns in lateral inhibition models [11]–[15]. These models can be viewed as simplified versions of juxtacrine signaling, in which the circuit inside each cell is reduced to a single protein concentration, whose effect is to inhibit the production of the same protein in neighboring cells. Such models can be pictorially represented on a hexagonal 2D lattice. It has been shown [11] how these models generically lead to regular patterns where the expression activity in each cell can be either in a high or low state, and on each row a repeated high-low-low pattern is found, so that each cell with a high activity is surrounded by silenced cells.

In this paper, we show that the aforementioned pattern is not always achievable and the pattern obtained depends on how the tissue is formed. We consider two mechanisms of tissue growth: (i) through cell division where a mother cell gives birth to a daughter cell in each time step of the growth; and (ii) through cell migration where cells migrate towards the tissue and attach to its boundaries.

The main objective of this paper is to address the following general points: 1) Whether it is possible to obtain a regular pattern in a dynamically growing tissue; 2) Whether it is possible to obtain a regular pattern in a large group of non-differentiated cells; 3) The requirements of regular pattern formation, including the role of growth speed; 4) Robustness of the patterns to mutations; 5) Sensitivity of the pattern to mutations during tissue formation.

## Results

### Tissue Formation

The growth and remodeling of a tissue depends on the history of its mechanical environment as well as on genetic and epigenetic factors. There are two fundamental ways of how new cells are added to the existing tissue. Generally, growth of hard tissues occurs by adding of material onto the existing surface, whereas soft tissues grow by adding material internally [16]. In the following, we will explore four simple algorithms of forming the tissues using the model described in the methods section. These algorithms capture basic features of the different growth modes present in real tissues, and provide a proof of principle that the outcome of cell to cell interaction may dramatically depend on how the tissue is generated.

**Figure 1:**
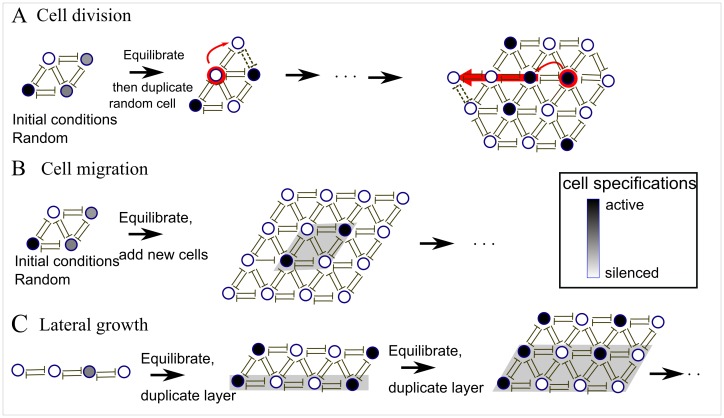
Three different growth mechanisms of tissues on hexagonal lattices. A). Cell division: A random cell in the tissue is doubled in each time step. The daughter cell is born in the same state as the mother cell and existing cells are pushed outwards (in a random direction) by the newly born cell. After each cell cycle the entire tissue is equilibrated by the dynamical equations (eq. 2). B). Cell migration: Cells are in each time step migrated onto the entire boundary of the tissue. All newly arriving cells are in the silenced state. The tissue is equilibrated between each growth step. C). Lateral growth: the tissue is grown only along one side through cell divisions, where each daughter cell is born in the same state as the mother. Between each addition of a new layer, the entire tissue is equilibrated. The parameter values were: 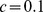, 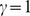, 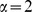, 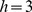, 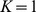.

- Through *cell division*: The system is initiated by 4 cells in random initial states, which may range from silenced to active. The cell cycle proceeds in two steps: protein activities in the cells are evolved to equilibrium using the dynamical equations (1) and (2). Then, one of the cells, chosen at random, is doubled. The new daughter cell has the same activity as the mother cell. The new cell is placed in a randomly chosen site that is adjacent to the mother cell, and the row of existing cells in that direction is pushed outwards. The tissue with one new cell is now equilibrated after which a new cell is doubled, and so on (Figure 1).
- Through *cell migration*: In each step, a new layer of silenced/inactive cells is added adjacent to all existing tissue cells after which the tissue is dynamically equilibrated by the dynamical equations, Figure 1.
- Through *lateral growth*: New cells are added layer by layer in one direction only, starting with one row of cells. The new row of daughter cells is obtained through cell division from the existing mother cells of the previous row, Figure 1C. After the growth of a new layer, the tissue is equilibrated.
- Through a *random configuration of cells* in silenced and active states: In this case, all cells are present in the tissue from the beginning of the simulation and the tissue does not grow. Each cell is initiated in a random state taken from a broad range of values from low (silenced) to high (active). After initiation, the entire tissue is equilibrated into the final state.

### Patterns of Cell States Depend on How the Tissue is Generated

**Figure 2:**
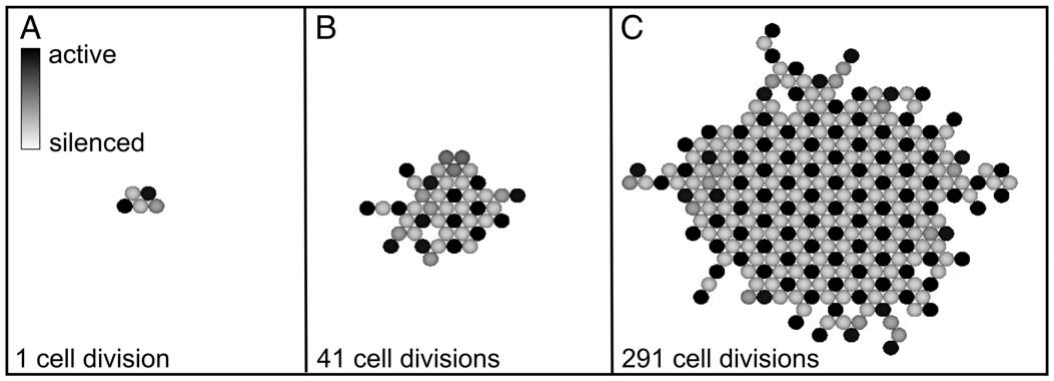
Tissue growth under cell division where in each cell cycle, a random cell is chosen and multiplied such that the daughter cell is born in the same state as the mother cell. The new cell pushes existing states in a random direction. The cells are graded from black (active cell) down to light gray (silenced cell). Note that the center of the tissue becomes completely ordered, i.e. each active cell is surrounded by six silenced cells.

The four ways of building the tissue produce different patterns of silenced and active cells. Figure 2 shows a tissue grown by the cell division method A), where one cell is doubled in each time step. After 291 divisions (Figure 2C), the center of the tissue is completely ordered with every active cell (black) surrounded by six silenced cells (light gray). Conversely, each silenced cell is surrounded by three active and three silenced neighbors. As the tissue expands, the pattern becomes ordered apart from slight irregularities in proximity of the boundary. This regular pattern is a general feature of this system, as shown in [11].

**Figure 3:**
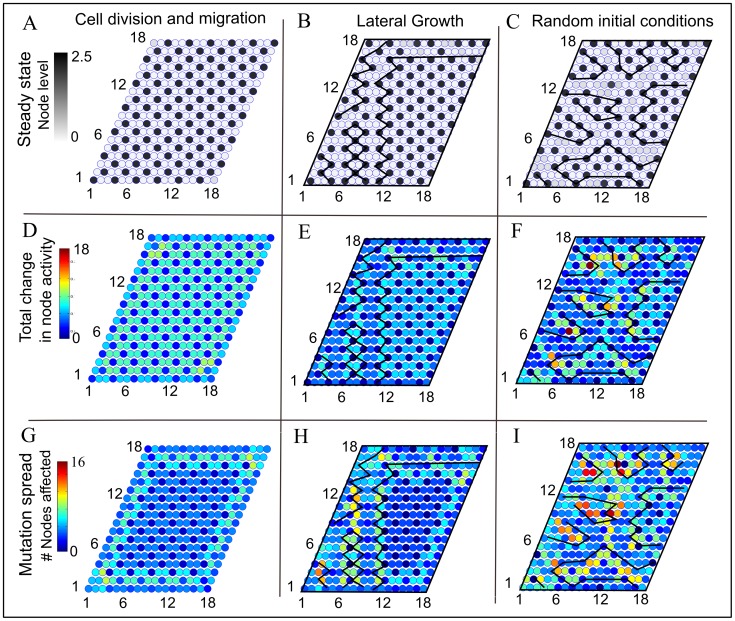
Total effect of mutations of single cells in the tissue. Starting from three different equilibrated tissues formed by cell division and migration (A), lateral growth (B) and random initial conditions (C), respectively, each cell of the tissue is mutated by the following rule: *silenced 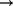 active* or *active 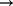 silenced*. The mutated cell is kept in the state it is set to after mutation. Panel (D–F): the color scheme indicates the sum of the activity changes over the entire tissue due to the mutation of one single cell. The lower panels (G–I) display the number of nodes affected by the mutation of the given cell. We note that the effects of mutations in the ordered tissue (A,D,G) are small. The tissues with disordered patches are very sensitive to mutations on the default lines separating these two phases. This is also apparent in the preformed tissue starting with cells being in random initial conditions where a larger effect occurs all along the default boundaries.

Generation of the tissue by the different methods described in Figure 2 lead to ordered and disordered patterns of cell activity. Cell division and migration, method A) and B) produces an ordered pattern (Figure 3A), both when the new cells are in an active or silenced state. In contrast, lateral growth results in random patches of disordered and ordered patterns in the tissue (Figure 3B). The boundary between ordered and disordered patterns (denoted default lines) are defined by the cells being in the high state which are not neighbored by two silent cells in any of the six directions of the hexagonal lattice. The positions of the default lines are not static, but tend to move during tissue growth. The disordered pattern has been observed to be defined vertical areas in the tissue, independent of the size of the tissue, and not the very disordered pattern produced by random initial conditions (Figure 3C).

### Computational Approach for Simulations of Mutations

We here perform independent mutations in every cell of the tissue, with the same equilibrated state of the entire tissue as the starting point. The state of the mutated cell is not subject to changes any more, but the remaining cells in the tissue are equilibrated again, after the perturbation in the mutated cell. This simulates the effects of mutations inactivating the components receiving signals from neighboring cells (becoming permanently active) or components responsible for sending signals to neighboring cells (becoming permanently inactive). We now analyze the robustness to mutations of single cells on the different tissue patterns described above. A mutation is performed by switching the activity of a single cell from silenced to active or from active to silenced, depending of the initial state of the cell.

The recording of the effects of a mutation is performed in two ways, measuring:

- The total change in cell activities in the entire tissue after the mutation of a single cell,
- The number of cells whose activity is affected more than 5% after a mutation

**Figure 4:**
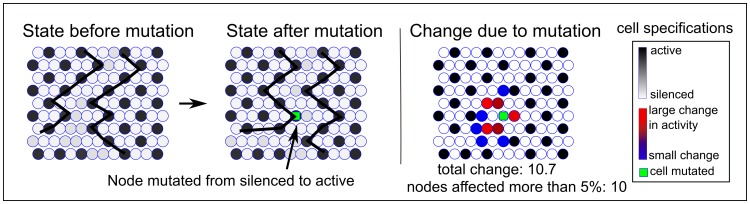
The mutation of a single cell in a tissue grown through cell division. On the default boundary between the ordered and disordered tissue (left panel), a silenced state is mutated into an active state (middle panel). The rightmost panel shows the effect of the mutation, where red symbolizes the largest effect and blue the smallest. The total number of cells that are affected more than 5% by the mutation is 10 (all colored).

Figure 4 shows an example of a mutation of one cell performed on an equilibrated tissue with a band of disorder at the center (Figure 4, left panel). On the boundary of the disordered patch (indicated by the ragged line) a cell is mutated from a silenced to an active state (middle panel). Figure 4 (right panel) shows the total distortion due to the mutation where the activity of 10 cells are affected more than 5% (the total distortion is estimated as the sum of the absolute values of activity changes). The color indicates which cells are affected the most (red) and which are affected the least (blue). This procedure is performed for each cell of the tissue.

### Ordered Patterns are More Robust Against Mutation of Cells than Disordered Patterns

The two lower rows (D–I) of Figure 3 shows the results of mutations in the three different tissues we consider: tissues grown by cell division or cell migration (Figure 3A), by lateral growth (Figure 3B) and a tissue spanned by cells having random initial conditions (Figure 3C). We conclude that.

- Ordered cell patterns are more robust against mutations than the disordered patterns
- Effects of mutations are more pronounced at the boundaries between the ordered and disordered patches.

The tissue in Figure 3A is ordered before mutations. In this case, the effect of mutations is very small and the only detectable effect is observed for the few cells close to the boundaries (Figure 3D,G) where the cells are not stabilized from all sides.

The tissue built by lateral growth (Figure 3B) shows clear boundaries between ordered and disordered patterns. We find that this boundary is the region where the state of the tissue is less robust against the effect of a mutation. The effects of mutations are more pronounced along the default lines in the pattern than within ordered patches. The same picture is seen, as well, for the last tissue made from cells with random initial conditions (Figure 3C).

The effect of mutations have been analyzed using statistical methods as those described in [17], [18]. In Table 1 we display the average number of cells affected (i.e. more than 5%) by a single cell mutation turning a silenced cell active or an active cell silenced, in order to quantify the effects of mutations in the lattice. The first two rows in Table 1 are averages due to mutations over the entire tissue (except close to boundaries to avoid boundary effects) where the top most row shows measurements for all cells that were silenced before the mutations and the second row shows measurements for all cells that were active before mutations. The ordered tissue grown by cell migration is the most robust to mutations. In particular, it is extremely robust to the class of mutations turning active cells silenced. Making a silenced cell active destabilizes other active cells close by, causing a rearrangement of the active and the silenced cells in the surroundings. However, mutating and keeping an active cell silenced, releases the repression of the neighboring cells and allows for a slight increase in protein activity. This latter effect is not as fatal for the pattern structure as introducing another active cell in the pattern.

**Table 1:** Average number of cells which are affected by more than 5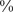 due to a single cell mutation of either a silenced to active cell, or an active to silenced cell, mean (standard deviation) in lattices with dimensions of 90×90 cells.

Tissue growth	Migration	Lateral	Random
Silenced to active, mean (std)	3 (0)	4 (1.8)	6.7 (2.8)
Active to silenced, mean (std)	0 (0)	0.7 (1.2)	2.8 (2)
**Cells mutated from silenced** **to active**			
less than 2 cells from a defaultline, mean (std)	–	5.2 (2.1)	6.8 (2.8)
2 or more cells from a defaultline, mean (std)	–	3.1 (0.5)	5.7 (2.0)
**Cells mutated from** **active to silenced**			
less than 2 cells from adefault line, mean (std)	–	1.8 (1.5)	3.1 (1.9)
2 or more cells from a defaultline, mean (std)	–	0.02 (0.2)	0.7 (1.1)

The upper two rows are for the entire tissue. The middle two rows display silenced cells mutated active, separated into two groups; cells located within two cells of a default line, and those two cells or more from the default line. The lower two rows are likewise for cells that are mutated from active to silenced. Only cells within a distance of 3 cells from the outer boundary of the tissue are included in these counts, to avoid boundary effects. A tissue built by migration has no defects and thus no counts in the 4 lower rows. The average number of cells is taken over all cells and does thus include cells that cause less than 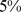 change (contributing as zero and thereby lowering the average).

The effect of mutating a cell from silenced to active is statistically larger (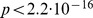) than when a cells mutated from active to silenced, in all tissues.

### Effects of Mutations are More Pronounced at the Boundaries Between the Ordered and Disordered Patches

For the tissues with disordered patches, we observe that the most pronounced effect of mutations occurs in cells located close to a default line (see Figure 3 E,F,H,I). With the aim of capturing this effect we separated the cells into two groups: those located within two cells from a default line and those beyond two cells (Table 1 lower rows), considering cells in both ordered and disordered areas of the tissue and measuring the shortest distance from the cell to the default lines in the tissue. We see that the effect of a mutation is significantly higher when performed within two cells of a default line in a tissue built by lateral growth, both when cells are mutated from silenced to active (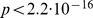) and when they are mutated from active to silenced (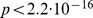). The effect of a mutation performed in the random tissue within two cells of the default line is additionally statistically higher than when the mutation is performed more than two cells away from the default line, both for cells mutated from silenced to active (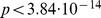) and cells mutated from active to silenced (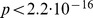).

Mutation of cells have a larger effects on the surrounding tissue in the completely random tissue, where defects dominate, than observed in the tissue built by lateral growth. This effect is due to the less organized boundary between the disordered and ordered patches in the random tissue (see Figure 3).

Generally, we can conclude that mutating a silenced cell to become active does always have an effect but the active cell must be close to the default line to have an effect on the state of the cells. Also the effect of the silenced cell mutated active is larger at the boundary of a default line.

### Dependence of Pattern Formation on Growth Speed

**Figure 5:**
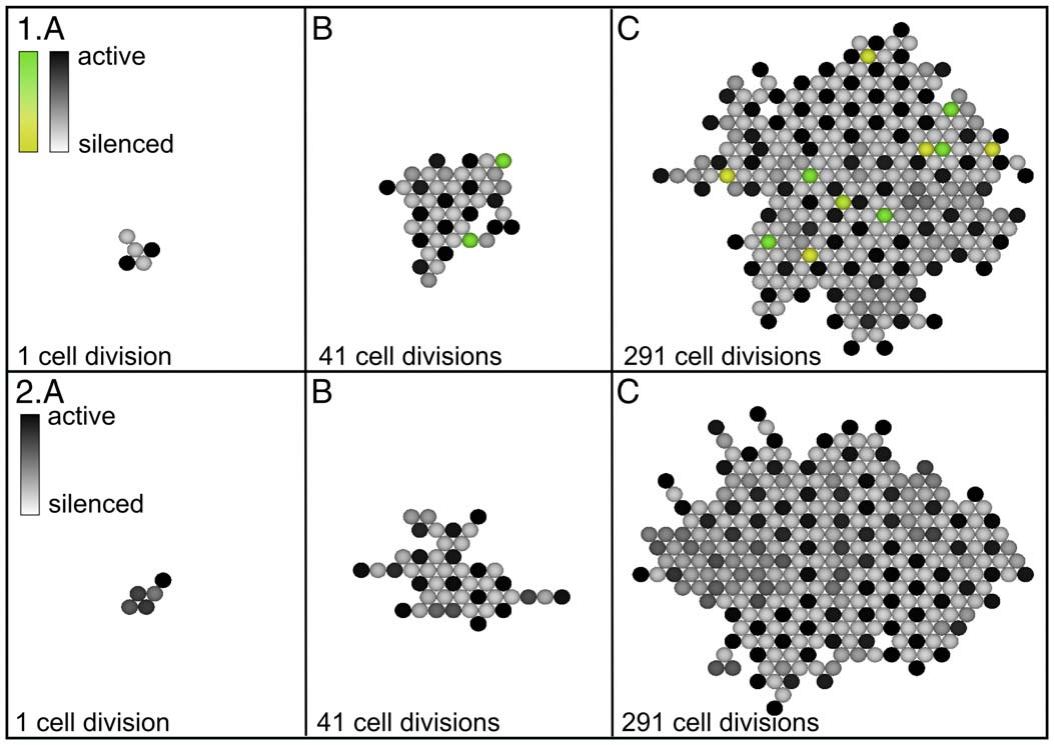
Disordered patches appear when cells are mutated during tissue growth and when grown with accelerated speed. (1A–C) Mutations during growth. In 5% of the cell divisions, the mother cell is mutated (and kept) in either a silenced (yellow) or active state (green) state. This creates a tissue with strongly disordered patches. (2A–C) Effect of growth speed on the activity pattern. The equilibration time 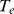 between each cell cycle is now of the size of the degradation time 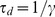 and is thus much shorter than the equilibration time used in Figure 2. Both disordered and ordered patches now appear side by side in the tissue.

Figure 5(1A–C) shows a growth series, similar to the one in Figure 2, with the difference that mutations are now made during growth. In 5% of the cell divisions, the mother cell is mutated (and kept) into either a silenced (yellow) or active state (green), which creates a tissue with strongly disordered patches. As the tissue expands, such mutations lead to a disordered tissue.

During growth via cell divisions, the tissue is equilibrated through the dynamical equations (2) after each cell cycle. Equilibrium is reached in a timescale mostly determined by the degradation time of the regulatory protein 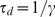 min. When the time between consecutive cell additions, 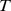 (typical time of cell duplication or cell migration), is much larger than the degradation time, the tissue can equilibrate faster than it grows, resulting in an ordered pattern.

The effect of variations in equilibration time on the tissue patterns is shown in Figure 5(2A–C), indicating three stages of the growth with a speed such that 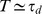. This relatively fast growth leads to a pattern that is disordered with patches where the cells equilibrate at an intermediate state.

## Discussion

In this paper we studied the possible causes of ordered and disordered patterns in the formation of tissues, and the stability of tissue patterns with respect to mutations. We have modeled various mechanisms for tissue formation during development with an underlying hexagonal symmetry. This is a natural ordering of cells that are closely packed in a single layer (see Figure 1) and we have assumed that all neighboring cells repress each other. Seen from a biological viewpoint, the most natural way to grow a tissue is through cell division. We find that this invariably leads to ordered patterns when cells are allowed to equilibrate before further cell division. Cell migration defines an alternative way of growth which also leads to ordered patterns in the bulk. In both cases, some disorder is observed close to the tissue boundaries. Lateral growth, where an entire row of cells is integrated into the tissue in each cell cycle, on the other hand, leads to disordered patterns with default lines penetrating the tissue.

We observe that the ordered patterns are much more robust and stable against mutations than the disordered ones. Mutations in ordered tissues only lead to localized disorder with almost no large scale effect. On the contrary, mutations have more pronounced effects in the disordered patterns, in particular when mutations are performed very close to the boundary lines. These observations have fundamental biological implications: tissues which exhibit patches of disordered cell patterns might be much more affected by mutations, leading to possible developmental malfunctions. We have furthermore observed that disordered patterns in the tissue also appear during enforced speed of cell divisions. This indicates that in order to achieve biologically healthy stasis for a tissue, the growth speed has a physical lower bound which is set by the degradation rates of regulators involved. This observation suggests that (i) systems of natural lateral inhibition should have actively degraded components and (ii) a faster degradation rate of the slowest degraded component allows a faster growth of the tissue without introducing errors in the pattern.

## Materials and Methods

### The Model

In our phenomenological model, the tissue is idealized, which means that as opposed to real tissues it is made up of cells occupying sites on a hexagonal lattice, not containing any cells with less or more sides. A cell located on a specific site on the lattice is labeled by its coordinates (m,n). We consider the activity of a regulatory protein in each cell, denoted 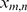, and assume that this activity can be repressed by signals received from the six neighboring cells. Cells with a low or zero cell activity are considered to be *silenced* and cells with a high cell activity are considered *active*. The dynamics of the protein activity of cell (m,n) is then given by the equation:
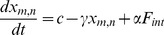
(1)


Here 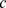 is the background level of protein production, 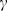 is the protein degradation rate, 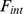 is the repression term from the neighboring cells and 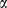 is the strength of this repression. We assume that repression can be modeled via a Michaelis-Menten term and that repression from different cells act multiplicatively, where a signal received from one neighboring cell is sufficient to trigger the repression of the protein level in a given cell. We prefer multiplicative coupling because in case of the additive coupling each neighbor cell would only contribute 1/6 of the repression and a threshold level would be needed. Additive repression does additionally require integration of signals received from the six neighboring cells [19]. The multiplicative function 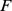 can be written as:
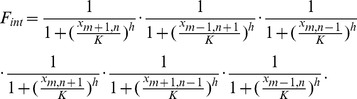
(2)


Here, 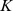 is the dissociation constant of the binding complex and 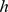 is the Hill coefficient measuring cooperativity. We assign the same parameters to all cell-to-cell interactions in the lattice. A typical set of parameter values for the simulations presented in the paper is: 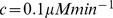, 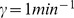, 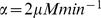, 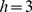, 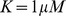.

In contrast to previous studies [20] where periodic boundary conditions were imposed, in all simulations presented in this paper we apply open boundary conditions meaning that 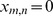 if node 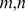 is not occupied by a cell.

Model simulations were performed in MatLab (version 7.11.0). Statistical analysis were performed in R [21] using a two-sided Wilcoxon rank sum test.
